# Limited Utility of Lymphocyte-Based Inflammatory Indices in Troponin-Negative Unstable Angina Pectoris

**DOI:** 10.3390/jcdd13050200

**Published:** 2026-05-08

**Authors:** Şükriye Uslu, Gülsüm Meral Yılmaz Öztekin, Ahmet Genç, Ekin Can Çelik, Şakir Arslan

**Affiliations:** 1 Department of Cardiology, Antalya Training and Research Hospital, Health Science University, Antalya 07100, Turkey; gmeralyilmaz@gmail.com (G.M.Y.Ö.); gencahmet@yahoo.com (A.G.); drsakirarslan@gmail.com (Ş.A.); 2 Department of Cardivascular Surgery, Antalya Training and Research Hospital, Health Science University, Antalya 07100, Turkey; ekincancelik@gmail.com

**Keywords:** unstable angina, acute coronary syndrome, Systemic Immune–Inflammation Index (SII), lymphocyte-based inflammatory indices, coronary artery disease severity

## Abstract

Background: This study investigated the role of lymphocyte-based inflammatory indices (LBIIs) in predicting severe coronary artery disease (CAD) in patients undergoing coronary angiography (CAG) for unstable angina pectoris (USAP). Methods: Records of patients who underwent CAG between January 2023 and December 2024 were retrospectively reviewed. The patients were divided into two groups based on coronary artery stenosis severity: non-severe CAD (<70% stenosis) and severe CAD (≥70% stenosis). Demographic data, risk factors, and complete blood count parameters were recorded. Six LBIIs were calculated: the neutrophil-to-lymphocyte ratio (NLR), monocyte-to-lymphocyte ratio (MLR), platelet-to-lymphocyte ratio (PLR), Systemic Immune–Inflammation Index (SII), Systemic Inflammatory Response Index (SIRI), and Systemic Immune–Inflammatory Response Index (SIIRI). Diagnostic performance was evaluated using logistic regression and ROC curve analyses. Results: Out of 505 patients, 234 (46.3%) had severe CAD. Among the six LBIIs, only the SII differed significantly between groups in univariate analysis and showed moderate discrimination in the ROC analysis (AUC 0.71; 95% CI 0.661–0.762; *p* < 0.001; sensitivity 76.2%; specificity 56.1%). However, the SII was not an independent predictor in the multivariate analysis. Conclusions: LBIIs (NLR, MLR, PLR, SII, SIRI, and SIIRI) do not provide a clinically significant and independent contribution to predicting severe CAD in USAP patients undergoing CAG. Although the SII performed moderately well in the univariate analysis, it lost independence in the multivariate analysis and is thus not suitable for use as a standalone marker in clinical decision-making.

## 1. Introduction

Coronary artery disease (CAD) continues to be one of the leading causes of morbidity and mortality worldwide. Coronary artery disease occurs when plaques that develop in the artery walls as a result of atherosclerosis progress and restrict blood flow. Symptoms generally appear when the plaques reach an advanced stage [1].

Unstable angina pectoris (USAP), a major component of acute coronary syndromes (ACSs), is defined as transient myocardial ischemia without evidence of significant myonecrosis [2]. Cardiac tests commonly used on admission have limited discriminatory power in identifying patients at risk for obstructive CAD [3]. Although a low in-hospital event rate is observed, the 1-year incidence of cardiac events is high (26%), particularly in those with ACSs [4]. The lack of a revised diagnostic and etiological algorithm for patients with USAP remains challenging for clinicians.

Recent studies have indicated that lymphocyte-based inflammatory indices (LBIIs) derived from whole blood parameters may be predictive of lesion severity, major adverse cardiovascular event (MACE) rates, and mortality in CAD [5,6,7,8,9,10,11]. These studies evaluated different patient populations together (stable CAD, USAP, non-ST elevation myocardial infarction (NSTEMI), and ST elevation myocardial infarction (STEMI)), and no analysis specific to USAP patients was performed, making generalization of the results to USAP patients difficult.

While systemic inflammatory markers have demonstrated utility in predicting complications in some vascular conditions (e.g., restenosis after carotid intervention), emerging evidence suggests that their predictive value may be context-dependent and dependent on the underlying inflammatory phenotype [12]. Specifically, inflammation in atherosclerosis is increasingly recognized as spatially heterogeneous and often localized at the plaque level, which may not be adequately captured by circulating biomarkers alone. This is particularly relevant in troponin-negative USAP, where plaque erosion may predominate and be characterized by localized rather than systemic inflammatory responses.

When the inflammatory processes in atherosclerotic plaques in acute coronary syndrome are evaluated, plaque erosion is seen more frequently than plaque rupture in USAP [13,14]. Plaque erosion exhibits a lower inflammatory profile and localized immune response [14,15]. In addition, USAP is associated with preserved coronary flow, smaller infarct size, and lower levels of myocardial necrosis markers [13,16,17].

In this study, we evaluated the hypothesis that LBIIs may not adequately reflect the low-grade, localized inflammation seen in troponin-negative USAP, particularly if plaque erosion predominates in this population. Therefore, we aimed to investigate the role of LBIIs (neutrophil-to-lymphocyte ratio (NLR), monocyte-to-lymphocyte ratio (MLR), platelet-to-lymphocyte ratio (PLR), Systemic Immune–Inflammation Index (SII), Systemic Inflammatory Response Index (SIRI), and Systemic Immune–Inflammatory Response Index (SIIRI)) in predicting CAD severity in USAP patients who underwent coronary angiography (CAG).

## 2. Materials and Methods

This retrospective, cross-sectional study was conducted at a single center between January 2023 and December 2024. During the study period, 3529 patients underwent coronary angiography at our institution. Among patients presenting with acute coronary syndrome, 997 had STEMI (28%), 1744 had NSTEMI (49%), and 594 had USAP (17%), with the remaining 194 (6%) undergoing angiography for other indications (e.g., myocarditis, newly diagnosed heart failure etiology evaluation). Among the 594 USAP patients, 505 met all inclusion and exclusion criteria and were included in the final analysis.

Patients over 18 years of age, those diagnosed with USAP according to the 2025 ACC/AHA definition, and those with available complete blood count parameters were included in the study. Serum troponin levels were measured using a chemiluminescence method with the MAGLUMI X3 automated immunoassay analyzer (Snibe Diagnostics, Shenzhen, China). Troponin levels were evaluated at presentation (0 h) and at 3 h after admission to the emergency department. Patients were classified as troponin-negative if both measurements remained below the 99th percentile upper reference limit (100 ng/L for hs-TnT). Patients with either measurement ≥ 100 ng/L were excluded from the study. Both baseline and follow-up ECG findings were evaluated for the presence of ST-segment changes, T-wave inversions, and other dynamic changes consistent with acute ischemia.

Patients with severe kidney disease (estimated glomerular filtration rate [eGFR] < 30 mL/min/1.73 m^2^, calculated using the Modification on Diet in Renal Disease (MDRD) equation), liver disease, white blood cell (WBC) count <4000 cells/µL, hematologic diseases (including anemia with hemoglobin level below 10 g/dL), autoimmune diseases, severe valvular heart disease, active cancer, thyroid dysfunction, and clinically significant acute or chronic inflammation, and patients receiving steroid therapy were excluded from the study. These exclusion criteria were designed to minimize confounding from conditions that could independently affect lymphocyte-based inflammatory indices. The application of these criteria resulted in the exclusion of 89 USAP patients (15% of the initial USAP cohort), thereby reducing selection bias by creating a homogeneous cohort with stable baseline inflammatory parameters.

Severe CAD was defined as greater than 70% obstruction in at least one main coronary artery (or at least 50% obstruction in the left main coronary artery).

Complete blood counts and biochemical parameters were recorded for all patients. Lymphocyte-based inflammatory indices were calculated as follows:

Neutrophil-to-Lymphocyte Ratio (NLR): Neutrophil count/Lymphocyte count.

Monocyte-to-Lymphocyte Ratio (MLR): Monocyte count/Lymphocyte count.

Platelet-to-Lymphocyte Ratio (PLR): Platelet count/Lymphocyte count.

Systemic Immune–Inflammation Index (SII): (Neutrophil count × Platelet count)/Lymphocyte count.

Systemic Inflammatory Response Index (SIRI): (Neutrophil count × Monocyte count)/Lymphocyte count.

Systemic Immune–Inflammatory Response Index (SIIRI): (Neutrophil count × Monocyte count × Platelet count)/Lymphocyte count.

The study protocol for this study was approved by the local research ethics committee, and it complies with the Declaration of Helsinki.

### Data Analysis

The results are reported as mean ± SD, median (25th to 75th percentile), or n (%). We assessed missing data and skewness using SAS version 9.4 (SAS Institute Inc., Cary, NC, USA), with normality analyzed through boxplots.

Groups were compared using a chi-square test or *t*-test for normally distributed data (or their non-parametric equivalent for skewed data). The bivariate correlation between inflammatory variables was assessed using Pearson’s r. Association with the primary study outcome was evaluated using binary logistic regression. Each predictor was assessed for its association with the primary outcome (stenosis < 70% or stenosis ≥ 70%) using univariate and multivariate logistic regression. Predictors with a *p*-value *<* 0.05 in the univariate analysis were included in subsequent multivariate logistic regression modeling. The final model retains only those variables that are statistically significant and contribute meaningfully to explaining variability in the primary outcome. The receiver operating characteristic (ROC) curve was utilized only for variables that showed statistical significance in the univariate analysis. Youden’s index was used to determine the optimal cutoff values of the SII. A two-sided *p*-value of <0.05 indicated statistical significance. Bootstrap validation was performed with 500 resamplings using SAS/STAT^®^ v. 9.4 procedures (PROC PLM RESTORE) for model validation.

## 3. Results

Table 1 shows the baseline characteristics of the study sample. The sample included 505 patients who underwent CAG and met the inclusion criteria. The mean age was 57.9 ± 11.12 years, and 377 (74.7%) patients were male. Hypertension (HT) was present in 55.6% of the patients, and 37.3% had diabetes mellitus (DM). When comparing the groups, patients with severe CAD were older and had a more unfavorable cardiovascular risk profile, and there was a higher proportion of males in this group. Differences in glucose, creatinine, LDL cholesterol, and EF were also evident in this group.

**Figure 1:**
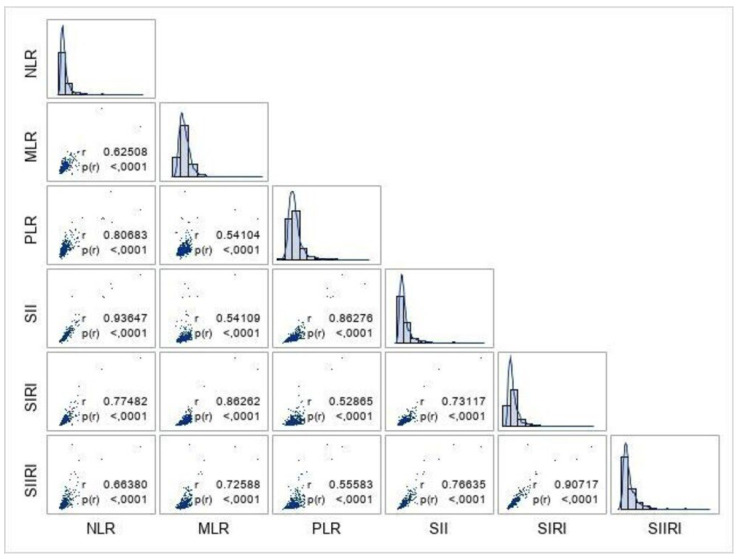
Distribution and cross-correlation between the inflammatory markers. The figure presents the distribution and pairwise cross-correlations between several inflammatory markers: neutrophil-to-lymphocyte ratio (NLR), monocyte-to-lymphocyte ratio (MLR), platelet-to-lymphocyte ratio (PLR), Systemic Immune–Inflammation Index (SII), Systemic Inflammatory Response Index (SIRI), and Systemic Immune–Inflammatory Response Index (SIIRI).

Figure 1 shows the distribution of the LBIIs in the study sample and their cross-correlations. Most inflammatory markers were slightly positively skewed, suggesting that most patients had low inflammatory burden, with only a few patients experiencing a significant burden. In addition, the figure shows that there was a moderate-to-strong positive correlation between all inflammatory biomarkers, with a near-perfect positive correlation observed between the NLR and SII (*r* = 0.9365, *p* = 0.0001), NLR and PLR (*r* = 0.8068, *p* = 0.0001), MLR and SIRI (*r* = 0.8626, *p* = 0.0001), PLR and SII (*r* = 0.8628, *p* = 0.0001), and SIRI and SIIRI (*r* = 0.9072, *p* = 0.0001). This indicates potential associations between these markers in predicting severe CAD.

Table 2 describes the univariate and multivariate associations between baseline characteristics and the study outcome. As can be seen from the results of the univariate analysis, compared with those with stenosis < 70%, patients with stenosis ≥ 70% were older, were more likely to be male, and had more prevalent cardiovascular risk factors, DM, HT, revascularization, glucose, creatinine, EF, and LDL. Among the six LBIIs, only the SII showed a statistically significant association with severe stenosis in univariate logistic regression (OR 1.00, 95% CI 1.00–1.00, *p* = 0.026). It is noteworthy that the SII did not show a significant difference between groups (Table 1, *p* = 0.106), but it reached statistical significance in the logistic regression analysis.

Age, gender, HT, DM, revascularization history, ejection fraction, glucose, creatinine, LDL cholesterol, and the SII, one of the inflammatory indices, were found to be significant in predicting severe stenosis in the univariate logistic regression analysis. However, the multivariate logistic regression analysis showed that the SII was not an independent predictor of severe stenosis (*p* = 0.229). After adjusting for traditional risk factors in the multivariate analysis, female sex emerged as an independent protective factor against severe stenosis (OR 0.3, 95% Cl 0.2–0.6, *p* < 0.007), indicating a 70% risk reduction compared with the male sex. The Hosmer–Lemeshow test indicated adequate model fit (chi-square = 5.004, *p* = 0.7575), confirming that the multivariate model appropriately fits the data. In order to test for multicollinearity in the final multivariate logistic regression analysis, the variance inflation factor (VIF) was calculated for the model. Since all VIF values were around 1, and highly correlated biomarkers, namely NLR, MLR, PLR, SIRI, and SIIRI, were not included in the model, it is assumed that multicollinearity was not present in the model. Interaction terms between SII and traditional risk factors (age, sex, DM, HT, ejection fraction, glucose, creatinine, and LDL cholesterol) were tested in the multivariate model. None of these interaction terms were statistically significant, and they were therefore dropped from the final model.

**Figure 2:**
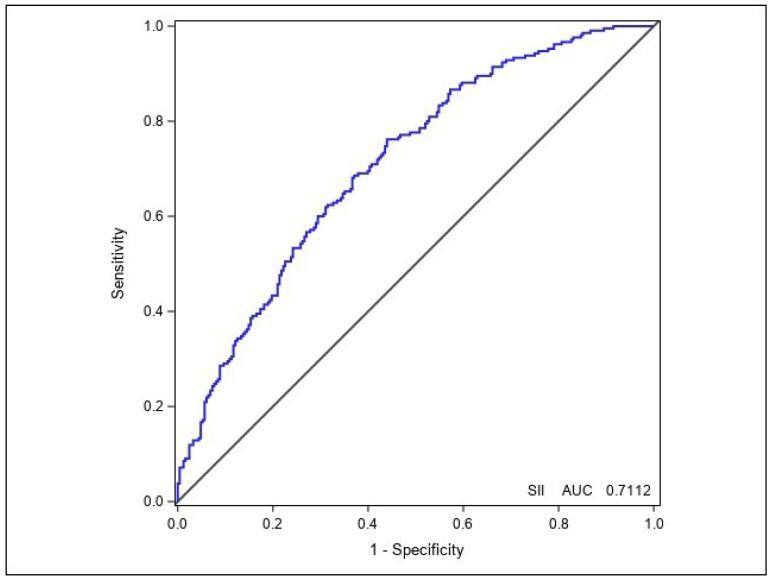
Receiver operating characteristic (ROC) curve for the Systemic Immune–Inflammation Index (SII).

The results of the ROC analysis for the SII are summarized in Table 3. The ROC analysis for the SII revealed an AUC of 0.7112 [95% CI: 0.661–0.762] (*p* < 0.001) (Figure 2). The results also showed a cutoff value of 1858.78 for the SII; it predicted stenosis with 76.2% sensitivity and 56.1% specificity.

### Model Validation

The analysis yielded an expected optimism of 0.019 for the c-statistic (AUC), with optimism-corrected c-statistic values of 0.652 (original) and 0.633 (corrected), respectively. The minimal difference between the original and optimism-corrected AUC values (0.003) indicates that the model fits the data well and is robust against overfitting, demonstrating good discriminative performance of the combined model.

## 4. Discussion

In this study, we evaluated the predictive value of LBIIs (NLR, MLR, PLR, SII, SIRI, and SIIRI) in severe coronary artery stenosis in our homogeneous group of troponin-negative patients with USAP.

Although the SII’s cutoff value of 1858.78 showed acceptable sensitivity (76.2%) for identifying patients with severe coronary stenosis (≥70%), its low specificity (56.1%) limits its clinical utility as a standalone predictive marker. However, in the logistic regression analysis examining the prediction of severe stenosis, the SII appeared to have significant predictive value in the univariate analysis, but it was not an independent predictor in the multivariate analysis. These analyses clearly demonstrated that these six lymphocyte-based indices did not provide additional benefit in predicting severe stenosis before CAG in the USAP patient group. Furthermore, the moderate-to-strong positive correlations among all LBIIs suggest that there are potential relationships between these LBIIs. However, traditional risk factors such as age, sex, ejection fraction (EF), and LDL cholesterol were found to be predictors of the presence of severe CAD.

The reversal of the association between sex and severe stenosis from the univariate to the multivariate analysis is of particular note. While male sex was a risk factor in the initial analysis, female sex emerged as an independent protective factor after adjusting for other comorbidities. To test for Simpson’s paradox, we performed interaction analyses testing interaction terms between SII and multiple variables (age, sex, DM, HT, ejection fraction, glucose, creatinine, and LDL cholesterol). These interaction terms were not statistically significant (*p* > 0.05), confirming that the paradoxical sex reversal cannot be fully explained by differential comorbidity effects alone. However, given the exploratory nature of this study, formal confirmation in larger, prospective cohorts would be valuable. Once these variables were statistically controlled, the independent, protective association of female sex became evident. This underscores the critical importance of multivariable adjustment in interpreting sex-specific cardiovascular risk and suggests that the impact of sex on CAD severity is deeply intertwined with the burden of other traditional risk factors.

Acute coronary syndromes are divided into STEMI and NSTEMI, and NSTEMI patients with negative troponin are defined as USAP [2]. In patients with chest pain, negative cardiac biomarkers, hemodynamic stability, and normal ECG, there is always a diagnostic dilemma, and the guidelines recommend repeating tests after a certain period of time [2]. Parameters that can predict the severity of coronary artery stenosis in this patient group need to be investigated and validated.

Coronary artery disease occurs as a result of plaque buildup in the coronary arteries during a process called atherosclerosis [1]. When the initially asymptomatic process begins to cause symptoms, plaques usually grow in the coronary arteries and block blood flow to the myocardium [1].

In the literature, there are conflicting results in studies that have examined the effect of inflammatory indices on MACE rates in non-homogeneous patient groups (newly diagnosed CAD, ACS, or AMI patients) [5,6,7,8,9,10,11]. The main reason for these conflicting results is that different patient populations (USAP, NSTEMI, and STEMI) were evaluated together, and pathophysiological differences were ignored.

The inflammatory process in atherosclerotic plaques was evaluated in the review published by Cheng and Wang [18]. Pathology studies of plaques causing acute coronary thrombosis have shown three types of structural changes: rupture of the fibrous cap (in 55–65% of cases), endothelial erosion (in 30–35% of cases), or calcified nodule formation (in 2–7% of cases) [19]. Plaques with thin and ruptured fibrous caps (RFCs) have large necrotic cores, and the necrotic cores usually indicate severe inflammation [20]. The necrotic core is exposed upon rupture, interacting with the circulating blood and triggering thrombosis [20,21]. On the other hand, erosion occurs in plaques with thick and intact fibrous caps (IFCs) or plaques without necrotic cores [20,22]. These plaques have less inflammation, but loss of the endothelial layer leads to thrombosis at the eroded site [20,21]. After thrombotic events, plaques may heal by developing thick fibrous caps, which further narrow the arterial lumen and may cause re-rupture or erosion [18,20].

Although intravascular imaging studies have established OCT-based classification of plaque erosion and rupture, LBIIs, such as the SII, may fail to capture localized endothelial inflammation [14,15]. This observation suggests the potential value of imaging-based or molecular hybrid indices for risk stratification in USAP.

The literature suggests that RFC is the most common mechanism in STEMI patients, while IFC is more prevalent in NSTEMI patients [22,23]. OCT-based studies have demonstrated that RFC-ACS is associated with higher systemic inflammatory burden and worse clinical outcomes (two-year major adverse cardiac event (MACE) rate of 26.7% vs. 14.3% in IFC-ACS), with systemic activation of immune pathways including the IL-1β to IL-6 axis. In contrast, IFC-ACS patients exhibited a lower inflammatory profile with more localized immune responses [14,15]. If our troponin-negative USAP cohort is enriched for IFC phenotypes, this could explain the limited utility of systemic inflammatory indices in predicting severe CAD.

Scalise and colleagues demonstrated that systemic inflammatory indices effectively predicted restenosis after carotid intervention, suggesting that systemic inflammation plays a significant role in that setting [12]. However, our findings in troponin-negative USAP suggest a different pattern: if plaque erosion is characterized by localized rather than systemic inflammation, systemic biomarkers may inadequately capture the relevant inflammatory processes.

Patients with plaque erosion, more typically associated with the clinical presentation of NSTEMI and with milder inflammation, exhibit lower levels of myocardial necrosis markers, indicative of smaller infarct size and more stable presentations with preserved coronary flow [13,16,17]. We hypothesize that the lower systemic inflammatory burden in our troponin-negative USAP cohort reflects plaque erosion with localized rather than systemic inflammation, which would explain the limited utility of systemic inflammatory indices. Plaque erosion, in contrast to rupture-associated systemic immune activation, may involve primarily localized endothelial responses. This observation aligns with emerging evidence that atherosclerotic inflammation is spatially heterogeneous and often inadequately captured by circulating biomarkers. However, direct confirmation would require intravascular imaging (OCT or IVUS), which was not performed.

In the literature, the power of the NLR in predicting the severity of coronary artery stenosis in USAP patients was evaluated, with a reported sensitivity of 69% and specificity of 64% [24]. In our study, the AUC value of the SII was 0.71, with a sensitivity of 76% and a specificity of 56%. However, it was not found to be an independent predictor in the multivariate analysis. The reason for the lack of significant results across all LBIIs appears to be due to the limited inflammatory response mentioned in the pathophysiological mechanisms in USAP patients. Therefore, our findings suggest that risk stratification in USAP patients should be based on the clinical/ECG/dynamic biomarker strategies recommended by the guidelines [2]. However, given the exploratory nature of our study, it may be premature to conclude that LBIIs have absolutely no clinical utility. Future prospective studies are needed to evaluate whether these indices might provide incremental predictive value when integrated into comprehensive risk models or combined with novel imaging-based biomarkers.

## 5. Limitations

Our study has several limitations that should be acknowledged. First, this is a single-center, retrospective study. Retrospective data collection meant that our access to detailed examination and referral records during hospitalization was limited. Specific troponin values for individual patients were not reported, as all patients had values below the reference limit. We did not perform plaque characterization with OCT or IVUS, limiting our ability to directly test the hypothesis regarding plaque erosion versus rupture. Furthermore, due to the prolongation of the procedure, the need for technical expertise, and the increased cost, neither FFR (Fractional Flow Reserve) nor iFR (Instantaneous Wave-Free Ratio) was used; angiographic evaluation was based solely on anatomic criteria. The most significant limitation of our study is the lack of long-term follow-up data and MACE results, which limits the prognostic validity of the findings. Multicenter, prospective studies with external validation in independent cohorts are needed to confirm whether lymphocyte-based inflammatory indices retain their value in the low-inflammatory phenotype of ACS before clinical implementation. Specifically, such prospective validation is essential to confirm whether the lack of independent predictive value observed in our cohort holds true across diverse populations or if LBIIs might still offer clinical utility in specific subsets of USAP patients.

## 6. Conclusions

In patients with troponin-negative USAP, LBIIs (NLR, MLR, PLR, SII, SIRI, and SIIRI) are not independent and clinically adequate predictors of severe coronary stenosis. While the SII demonstrated moderate discrimination in the univariate analysis, it did not maintain independence in the multivariate analysis. This finding suggests that indices based on systemic inflammation may not adequately capture the localized endothelial processes underlying troponin-negative USAP. However, prospective studies are needed to validate these findings and determine whether LBIIs retain value when combined with established clinical models.

## Figures and Tables

**Table 1:** Baseline characteristics of the study sample.

	Total (N = 505)	Stenosis < 70% (N = 271)	Stenosis ≥ 70% (N = 234)	*p*-Value
Sex, male	377 (74.7%)	188 (69.4%)	189 (80.8%)	0.003
Age	57.9 (11.12)	56.1 (11.20)	59.9 (10.69)	0.001
Hypertension	267 (55.6%)	131 (50.8%)	136 (61.3%)	0.021
Diabetes Mellitus	177 (37.3%)	79 (31.0%)	98 (44.7%)	0.002
Smoking				0.060
Non-smoker	163 (35.2%)	98 (39.4%)	65 (30.4%)	
Current smoker	241 (52.1%)	117 (47.0%)	124 (57.9%)	
Former smoker	59 (12.7%)	34 (13.7%)	25 (11.7%)	
Revascularization History				0.014
None	356 (70.5%)	202 (74.5%)	154 (65.8%)	
Stent	141 (27.9%)	68 (25.1%)	73 (31.2%)	
CABG	8 (1.6%)	1 (0.4%)	7 (3.0%)	
Ejection Fraction, %	57.9 (7.80)	59.1 (7.02)	56.5 (8.40)	<0.001
Glucose, mg/dL	123.1 (65.61)	114.8 (54.04)	132.7 (75.86)	0.023
Creatinine, mg/dL	1.0 (0.19)	1.0 (0.18)	1.0 (0.20)	0.016
LDL cholesterol, mg/dL	119.6 (44.06)	115.3 (39.19)	124.7 (48.75)	0.063
WBC, 10^3^/mm^3^	9.2 (2.67)	9.1 (2.70)	9.3 (2.63)	0.126
HGB, g/dL	14.1 (1.55)	14.0 (1.56)	14.3 (1.54)	0.033
NLR	2.9 (2.92)	2.7 (2.15)	3.1 (3.61)	0.228
MLR	0.3 (0.16)	0.3 (0.17)	0.3 (0.15)	0.407
PLR	114.9 (64.67)	110.1 (52.28)	120.6 (76.33)	0.332
SII	714.8 (743.18)	643.4 (517.57)	797.8 (934.27)	0.106
SIRI	1.9 (1.89)	1.8 (1.80)	2.0 (2.00)	0.148
SIIRI	485.1 (517.37)	452.7 (453.70)	522.7 (581.49)	0.090

CABG: coronary artery bypass grafting; LDL: low-density lipoprotein; WBC: white blood cell; HGB: hemoglobin; NLR: neutrophil-to-lymphocyte ratio; MLR: monocyte-to-lymphocyte ratio; PLR: platelet-to-lymphocyte ratio; SII: Systemic Immune–Inflammation Index; SIRI: Systemic Inflammation Response Index; SIIRI: Systemic Immune–Inflammatory Response Index.

**Table 2:** Univariate and multivariate logistic regression analyses for predictors of severe coronary stenosis (≥70%).

	Stenosis ≥ 70%			
	Univariate Analysis		Multivariate Analysis	
Covariate	OR (95% CI)	*p*-Value	OR (95% CI)	*p*-Value
Sex	1.85 (1.22–2.81)	0.004	0.3 (0.2–0.6)	<0.001 *
Age	1.03 (1.02–1.05)	<0.001	1.0 (1.0–1.1)	0.002 *
Hypertension	0.65 (0.45–0.94)	0.021	0.8 (0.5–1.2)	0.233
Diabetes Mellitus	0.55 (0.38–0.81)	0.002	0.6 (0.4–1.0)	0.070
Smoking	0.90 (0.49–1.65)	0.738		
Revascularization History	0.66 (0.45–0.97)	0.032	0.8 (0.5–1.2)	0.282
Ejection Fraction	0.96 (0.93–0.98)	<0.001	1.0 (0.9–1.0)	0.037 *
Glucose	1.00 (1.00–1.01)	0.003	1.0 (1.0–1.0)	0.126
Creatinine	3.02 (1.17–7.79)	0.023	0.7 (0.2–2.3)	0.556
LDL	1.01 (1.00–1.01)	0.021	1.0 (1.0–1.0)	<0.001 *
NLR	1.05 (0.99–1.12)	0.129		
MLR	1.02 (0.34–3.00)	0.979		
PLR	1.00 (1.00–1.01)	0.073		
SII	1.00 (1.00–1.00)	0.026	1.00 (1.00–1.00)	0.229
SIRI	1.03 (0.94–1.14)	0.479		
SIIRI	1.00 (1.00–1.00)	0.137		

OR, odds ratio; CI, confidence interval; NLR, neutrophil-to-lymphocyte ratio; MLR, monocyte-to-lymphocyte ratio; PLR, platelet-to-lymphocyte ratio; SII, Systemic Immune–Inflammation Index; SIRI, Systemic Inflammatory Response Index; SIIRI, Systemic Immune–Inflammatory Response Index. * *p* < 0.05.

**Table 3:** ROC analysis results for Systemic Immune–Inflammation Index (SII).

	AUC	SE	95% CI		*p*	Cutoff	Sensitivity	Specificity
SII	0.7112	0.058	0.661	0.762	<0.001	1858.78	0.762	0.561

## Data Availability

The data presented in this study are available upon request from the corresponding author.
